# Is Oestradiol a Key Player in the Sex Differences in Innate Immunity Through Toll-like Receptor Activation?

**DOI:** 10.3390/cells15141257

**Published:** 2026-07-13

**Authors:** Alexandros Popotas, Anne Delbaere, Georges Casimir, Francis Corazza, Viviane De Maertelaer, Nicolas Lefèvre

**Affiliations:** 1 Laboratory of Pediatrics, Université Libre de Bruxelles (ULB), 1020 Brussels, Belgium; georges.casimir@hubruxelles.be (G.C.); nicolas.lefevre@hubruxelles.be (N.L.); 2 Laboratory of Translational Research, Université Libre de Bruxelles (ULB), 1020 Brussels, Belgium; francis.corazza@chu-brugmann.be; 3 Paediatric Emergency Department, Queen Fabiola Childrens University Hospital (Hôpital Universitaire des Enfants Reine Fabiola)—University Hospital of Brussels (Hôpital Universitaire de Bruxelles), 1020 Brussels, Belgium; 4 National Center for Pediatrics, Centre Hospitalier de Luxembourg, 1210 Luxembourg, Luxembourg; 5 Fertility Clinic, Department of Obstetrics and Gynecology, University Hospital of Brussels (Hôpital Universitaire de Bruxelles—H.U.B), CUB Hôpital Erasme, Université Libre de Bruxelles (ULB), 1070 Brussels, Belgium; anne.delbaere@hubruxelles.be; 6 Laboratory of Immunology, Centre Hospitalier Universitaire (CHU) Brugmann, Université Libre de Bruxelles (ULB), 1070 Brussels, Belgium; 7 Statistical Unit, Institut de Recherche Interdisciplinaire en Biologie Humaine et Moléculaire (IRIBHM), Faculty of Medicine, Université Libre de Bruxelles (ULB), 1070 Brussels, Belgium; viviane.de.maertelaer@ulb.be; 8 Department of Pulmonology, Allergology and Cystic Fibrosis, Queen Fabiola Childrens University Hospital (Hôpital Universitaire des Enfants Reine Fabiola)—University Hospital of Brussels (Hôpital Universitaire de Bruxelles), 1020 Brussels, Belgium

**Keywords:** oestradiol, oestrogens, Toll-like receptors, innate immunity, sex differences, X chromosome, cytokine production

## Abstract

**Highlights:**

**What are the main findings?**

- A marked rise in oestradiol during IVF-induced ovarian hyperstimulation had little effect on TLR expression, NF-κB/MAPK signalling, or cytokine production in whole blood.
- Only TLR2/6-driven IL-6 and IL-10 responses increased during oestradiol exposure, while all other TLR-mediated cytokine responses remained stable.

**What are the implications of the main findings?**

- High circulating oestradiol alone is insufficient to explain sex differences in innate immune responses.
- Genetic mechanisms, particularly X-linked immune pathways, may play a greater role than sex hormones in shaping sex-specific immunity.

**Abstract:**

Research has shown a sex-specific immune response, with males having a worse prognosis in acute inflammatory diseases. While these disparities were initially attributed to sex hormones, increasing evidence points to a predominant role for X-linked genetic factors. Toll-like receptors and several components of their signaling pathway are encoded on the X chromosome and may contribute to these differences. We investigated whether increase in circulating oestradiol influences TLR-dependent immune response. Sixteen women undergoing controlled ovarian hyperstimulation for in vitro fertilisation were studied. Whole blood collected before treatment, during stimulation and at ovulation triggering was stimulated with ligands targeting TLR2/6, TLR1/2, TLR4 and TLR7/8. TLR2, TLR4 and CD99 expression, intracellular phosphorylated NF-κB p65, ERK1/2 and p38 MAPK, and cytokine production were assessed. Oestradiol levels increased markedly during treatment (48.1 to 1819.5 pg/mL; *p* < 0.001). Despite this rise, no or minimal impact on TLR2/4 and CD99 expression, intracellular signalling or cytokine release was detected. Only IL-6 and IL-10 in response to TLR2/6 stimulation increased significantly, with IL-6 positively associated with oestradiol variation. These findings indicate that oestradiol exerts a limited influence on TLR-dependent immune responses, supporting our view that sex-based immune differences are driven primarily by genetic rather than hormonal factors.

## 1. Introduction

Sex differences in the immune response to acute inflammatory conditions have been extensively documented, with males generally demonstrating a worse prognosis in cases of acute inflammatory diseases [1,2,3,4]. The previously attributed influence of sex hormones, particularly oestrogens, on the immune system has now been supplemented by studies drawing attention towards X-linked genes as contributors to this imbalance between men’s and women’s expression of specific immunity-related genes [5,6,7].

The innate immune response mediated by Toll-like receptors (TLRs) is subject to genetic control by loci situated on the X chromosome. X-linked genes play a crucial role in the regulation of TLR7, TLR8, and key components of the nuclear factor-kappaB (NF-κB) signalling pathway, such as IRAK-1, NEMO and BTK [8,9,10,11,12]. The cluster of differentiation (CD)99 protein, which is involved in leukocyte diapedesis, is encoded by a gene located in close proximity to the boundary between the pseudoautosomal region 1 (PAR1) and X-linked segments on the X chromosome [8,13].

The complexity surrounding these mechanisms contributes significantly to immune sex disparities, especially concerning TLR discrepancies, which may lead to a more diverse acute immune response among women [14].

This study aims to address knowledge gaps in sex-related immune differences, focusing on the hypothesis that chromosomal rather than hormonal factors play the dominant role. To test this, we examined TLR functions in whole blood from women undergoing controlled ovarian hyperstimulation, a model of supraphysiological oestrogen exposure that preserves immune responses in their native context. We hypothesised that, despite marked increases in circulating oestrogen, TLR signalling would remain largely unaffected, supporting the view that sex-based immune differences are primarily genetically determined.

## 2. Materials and Methods

### 2.1. Reagents

Highly purified lipopolysaccharide (LPS) from *Escherichia coli* 026:B6 was purchased from Sigma-Aldrich (St. Louis, MO, USA). Zymosan, PAM3CSK4 and Resiquimod (R848) were obtained from InvivoGen (San Diego, CA, USA). Monoclonal antibodies (mAbs) used for flow cytometry were acquired from BD Biosciences (San Jose, CA, USA). Roswell Park Memorial Institute (RPMI) 1640 medium containing L-glutamine was obtained from Westburg BV (Leusden, Netherlands). Penicillin–Streptomycin, fetal bovine serum (FBS) and phosphate-buffered saline (PBS) were purchased from Fisher Scientific (Waltham, MA, USA). Permeabilisation, fixation and flow-staining buffers and lysing solution were obtained from BD Biosciences (San Jose, CA, USA). The cytokine/chemokine magnetic bead-based panel was acquired from Merck Millipore (Darmstadt, Germany).

### 2.2. Subjects

Blood samples were collected from adult women recruited at the Fertility Clinic of Erasmus Hospital (Brussels, Belgium) during monitoring consultations of controlled ovarian hyperstimulation for in vitro fertilisation (IVF). Exclusion criteria included use of antithrombotic drugs, alcohol or tobacco, congenital or acquired immunodeficiency, haemodialysis, malignant cancer and HIV infection. The study received approval from the Erasmus Hospital ethics committee (reference: P2010/221), and participants provided written informed consent. Demographic and clinical data, including the type of ovarian hyperstimulation, were collected anonymously.

### 2.3. Blood Samples

Blood samples were obtained using venipuncture in 4 mL BD Vacutainer^®^ heparin tubes (sodium heparin REF 367869, BD Diagnostics, Franklin Lakes, NJ, USA). Withdrawals occurred on the first day of monitoring, just before starting ovarian stimulation, twice during cycle monitoring, and on the day of ovulation triggering. All experiments were performed at the laboratory of Pediatrics and the laboratory of translational research, Free University of Brussels, Belgium.

### 2.4. Laboratory Analyses

Whole blood was mixed in a ratio of 1:5 with RPMI 1640 medium containing L-glutamine, penicillin–streptomycin and 10% FBS and incubated, respectively, with RPMI (control), LPS (1 µg/mL), Zymosan (10 µg/mL), PAM3CSK4 (10 ng/mL) and Resiquimod (1 µg/mL) for 24 h in a humidified incubator at 37 °C with 5% CO_2_ (Heraeus HBB 2472b, Heraeus Instrument GmbH, Hanau, Germany). Supernatants were centrifuged (800× *g* for 5 min) and stored at −80 °C. Interleukin-1β (IL-1β), IL-6, IL-8, IL-10, IL-12(p70), Interferon-α2 (IFN-α2), IFN-γ and tumour necrosis factor-α (TNF-α) were measured by an Intelliflex platform (Merck, Darmstadt, Germany).

### 2.5. Expression of TLR2 and TLR4

Expression of TLR2 and TLR4 on neutrophils and monocytes was assessed by flow cytometry. Half of the whole blood was treated with RPMI, and the other half was stimulated with LPS at 1 µg/mL (60 min, 37 °C). Cells were treated with phycoerythrin (PE)-conjugated mAb against TLR4, fluorescein isothiocyanate (FITC)-conjugated mAb against TLR2, and allophycocyanin (APC)-conjugated mAb against CD14 (20 min, 4 °C). Control samples from the same patient were mixed with PE-conjugated isotype control for TLR4 and A488-conjugated isotype control for TLR2. Post removal of red blood cells with Fluorescence-activated cell sorting (FACS) lysing solution (BD Biosciences, San Jose, CA, USA), samples were analysed on a FACS Canto II flow cytometer (BD Biosciences, San Jose, CA, USA). Identification of leukocyte populations relies on side scatter (SSC) and CD14 expression criteria. After this procedure, detection levels of TLR2 and TLR4 were compared to those in the respective isotype control group.

### 2.6. Phosphorylation of Proteins of the TLR Signalling Pathway in Whole Blood

The intracellular levels of phosphorylated NF-κB p65, ERK1/2 and p38 MAPK in monocytes and polymorphonuclear cells (PMNs) were evaluated using flow cytometry. Each blood sample was divided into control (RPMI) and LPS-stimulated (10 µg/mL, 15 min, 37 °C) aliquots. Reactions were halted, and red blood cells were simultaneously removed using BD Phosflow Lyse/Fix Buffer (BD Biosciences, San Jose, CA, USA). Following a washing step, cells were permeabilised using BD Phosflow Perm Buffer and incubated on ice (30 min). Cells were treated with PE-conjugated mAb against phosphorylated NF-κB p65 (pS529), Alexa Fluor 488 (A488)-conjugated mAb against phosphorylated ERK1/2 (pT202/pY204), PE-cyanin 7 (Cy7)-conjugated mAb against phosphorylated p38 MAPK (pT180/pY182) and APC-conjugated mAb against CD33 (1 h, room temperature). Samples were assayed on a FACS Canto II. Leukocyte populations were differentiated based on SSC and CD33 expression.

### 2.7. Expression of Diapedesis Receptor CD99

Expression of the cell diapedesis receptor CD99 on PMNs, monocytes and lymphocytes was measured by flow cytometry. Whole blood was split for incubation with RPMI (control) or LPS (1 µg/mL, 60 min, 37 °C). Cells were treated with FITC-conjugated mAb against CD99 and APC-conjugated mAb against CD14 for 20 min at 4 °C. Control samples were mixed with FITC-conjugated isotype control for CD99. Post removal of red blood cells with FACS lysing solution, samples were analysed on a FACS Canto II. Identification of leukocyte populations relies on SSC and CD14. CD99 levels were compared with isotype controls.

### 2.8. Cytokines

Cytokine and chemokine concentrations in serum were measured using a Milliplex^®^ Map human cytokine/chemokine magnetic bead-based assay, according to the manufacturer’s instructions. The evaluated factors on the Intelliflex platform included IL-1β, IL-6, IL-8, IL-10, IL-12(p70), IFN-α2, IFN-γ and TNF-α.

### 2.9. Statistical Analysis

Categorical variables were summarised by their sample sizes (*n*) and percentages. Continuous variables were summarised by their means and standard errors. Successive multiple regressions were performed with a different immune response as the explanatory variable and the high oestradiol level, as well as age, as explanatory variables. Those were performed on the difference between the ovulation onset time and the time before treatment for both the explained variable and the high oestradiol level variable.

The different immune variables included cytokines IL-1β, IL-8, IL-10, TNF-α and IFN-α in whole blood; the intracellular quantity of the phosphorylated forms of NF-κB p65, ERK1/2 and p38 MAPK in the leukocyte population; the cell diapedesis receptor CD99 on PMNs, monocytes and lymphocytes; as well as expression of TLR2 and TLR4 on PMNs, monocytes and lymphocytes.

Non-parametric tests were applied for bivariate analyses. For comparisons across more than two related time points, the non-parametric Friedman test was employed. Wilcoxon signed-rank tests were used to assess intra-individual differences in immune markers (e.g., TLR2, TLR4, ERK1/2, NFκB, p38 MAPK, CD99) and cytokine expression between baseline and ovulation triggering. Correlations between changes in oestradiol (ΔE2) and changes in immune parameters (ΔMarker) were evaluated using Spearman correlation coefficients. Additionally, we assessed associations, including age as a potential confounding variable, with the use of multivariate linear regressions. Statistical significance was considered when *p* was <0.05. All statistical tests were two-sided. They were performed using IBM-SPSS (version 29.0.2) software (IBM Corp, Armonk, NY, USA).

## 3. Results

### 3.1. Study Population

A total of 16 patients were included in the study. Women with polycystic ovary syndrome (PCOS) or other major endocrine disorders known to significantly affect the hormonal milieu were not included in the study cohort. Among the included patients, 68.8% (*n* = 11) did not receive any pre-stimulation treatment, while 31.3% (*n* = 5) received oestradiol valerate 2 mg twice daily as pre-treatment. All patients were administered a gonadotropin-releasing hormone (GnRH) antagonist for pituitary inhibition during the protocol. Ovarian stimulation was achieved using recombinant Follicle-Stimulating Hormone (FSH) (*n* = 11) or urinary FSH (*n* = 5) (Table S1).

For ovulation triggering, 11 patients (68.8%) received recombinant human chorionic gonadotropin (hCG), 3 patients (18.8%) received a combination of recombinant hCG and triptorelin, 1 patient (6.3%) had triptorelin alone, and 1 patient (6.3%) was not triggered due to a gynaecological contraindication (Table S1).

The study population had a mean age of 33.2 years (range: 26–39 years) (Table S2).

### 3.2. Oestradiol Evolution During Ovarian Stimulation

**Figure 1:**
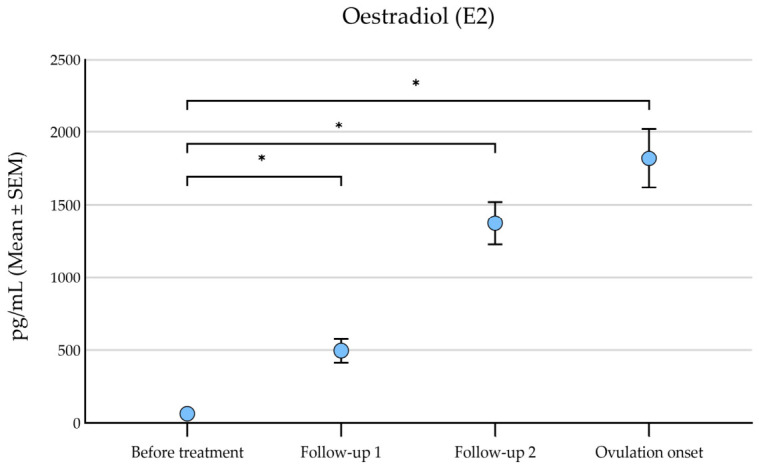
Oestradiol (E2) evolution during ovarian stimulation treatment. Data are presented as mean ± SEM. Significant differences are indicated by an asterisk (*). Serum E2 concentrations increased progressively during ovarian stimulation and were significantly higher at follow-up 1, follow-up 2, and ovulation onset compared with baseline (before treatment) (all *p* < 0.001, Wilcoxon matched-pairs signed-rank test, *n* = 16).

A significant increase in serum oestradiol (E2) levels was observed throughout the ovarian stimulation process. The mean E2 concentration before treatment was 62.9 pg/mL, compared to 1819.5 pg/mL at the time of ovulation onset (*p* < 0.001) (Figure 1).

**Figure 2:**
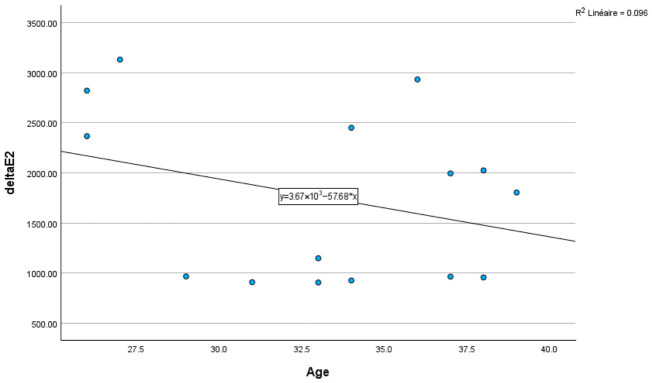
Scatter plot with linear regression line. Pearson’s correlation coefficient (r) and coefficient of determination (R^2^) are shown. No significant correlation was observed (*n* = 16).

Mean E2 values increased progressively across four measurement points: before treatment (62.9 pg/mL), first follow-up (403.6 pg/mL), second follow-up (1356.0 pg/mL) and ovulation onset (1819.5 pg/mL). A difference was found across time points (*p* < 0.001). No statistical correlation was found between age and the rise in oestradiol (ΔE2) (Figure 2).

### 3.3. TLR2 and TLR4 Expression in Immune Cells and Association with Oestradiol Response

#### 3.3.1. Controls

No changes were observed in the expression levels of TLR2 or TLR4 on neutrophils, monocytes or lymphocytes from the start of the treatment to the day of ovulation triggering (all *p* > 0.297). Multiple regression analyses adjusted for age showed no statistical association between changes in TLR expression (ΔTLR2 or ΔTLR4) and changes in oestradiol levels (ΔE2) across all cell subsets (all *p* > 0.450).

#### 3.3.2. LPS Stimulation

No differences were observed between baseline (before treatment) and ovulation onset for TLR4 expression on PMNs (*p* = 1.000), monocytes (*p* = 0.455) or lymphocytes (*p* = 0.368). Similarly, TLR2 expression showed no significant changes between the start of the treatment and the ovulation triggering day on PMNs (*p* = 0.305), monocytes (*p* = 0.553) or lymphocytes (*p* = 0.297). Multiple regression analyses adjusting for age revealed no statistical associations between changes in oestradiol levels (ΔE2) and changes in TLR4 or TLR2 expression (ΔTLR4 or ΔTLR2) in any of the studied cell populations (all *p*-values > 0.395).

### 3.4. ERK1/2 Expression in Immune Cells and Association with Oestradiol Response

#### 3.4.1. Controls

The phosphorylated ERK1/2 levels in neutrophils (ERK-PMN) decreased from baseline (before treatment) to ovulation onset (*p* = 0.025). The change in ERK-PMN (ΔERK-PMN) was not statistically associated with the change in oestradiol levels (ΔE2), even after adjusting for age (*p* = 0.130 for ΔERK-PMN; *p* = 0.093 for age).

No variation in phosphorylated ERK1/2 expression was observed over time in monocytes (ERK-M, *p* = 0.117) or lymphocytes (ERK-L, *p* = 0.252). Similarly, changes in ERK-M and ERK-L were not associated with ΔE2 in multivariate models adjusted for age (*p* = 0.840 and *p* = 0.493, respectively).

#### 3.4.2. LPS Stimulation

A decrease in the expression of the phosphorylated form of ERK1/2 was observed in PMNs between baseline and ovulation onset (*p* = 0.025). No changes were found in phosphorylated ERK1/2 levels in monocytes (*p* = 0.117) or lymphocytes (*p* = 0.252) over the same period. Correlation analyses revealed no statistical association between the change in ERK1/2 (ΔERK_LPS) and the change in E2 levels (ΔE2) for any cell type, even after adjusting for age (PMNs: *p* = 0.431; monocytes: *p* = 0.859; lymphocytes: *p* = 0.584).

### 3.5. NF-κB p65 Expression and Association with Oestradiol Response

#### 3.5.1. Controls

No change in NFκB p65 expression was observed between baseline (before treatment) and ovulation onset in neutrophils (NFκB p65-PMN, *p* = 0.597), monocytes (NFκB p65-M, *p* = 0.472) or lymphocytes (NFκB p65-L, *p* = 0.495).

In regression analyses adjusted for age, the change in the level of phosphorylated NFκB p65 in monocytes (ΔNFκB-M) was negatively associated with the change in oestradiol levels (ΔE2) (*p* = 0.039). No such association was found for neutrophils (*p* = 0.076) or lymphocytes (*p* = 0.113).

#### 3.5.2. LPS Stimulation

No changes in NF-κB p65 phosphorylation were observed in PMNs, monocytes or lymphocytes following LPS stimulation between baseline (T0) and T3 (PMNs: *p* = 0.528; monocytes: *p* = 0.860; lymphocytes: *p* = 0.753). Multiple linear regression analysis revealed no statistical association between the changes in the level of phosphorylated NF-κB p65 and changes in E2 levels (ΔE2) for any cell type after adjustment for age (PMNs: *p* = 0.078; monocytes: *p* = 0.164; lymphocytes: *p* = 0.147).

### 3.6. p38 MAPK Expression and Association with Oestradiol Response

#### 3.6.1. Controls

No changes were observed in the level of phosphorylated p38 MAPK between baseline and ovulation onset in PMNs, monocytes or lymphocytes. Multiple linear regression analyses showed no association between the change in oestradiol (ΔE2) and the change in the level of phosphorylated p38 MAPK expression (Δp38) for any cell type (*p* = 0.667 in PMNs, *p* = 0.925 in monocytes, *p* = 0.294 in lymphocytes). Age was also not a covariate in any of the models.

#### 3.6.2. LPS Stimulation

No change was observed in phosphorylated p38 MAPK (p38) expression in immune cell subsets following LPS stimulation between baseline and ovulation onset (*p* > 0.433 for all cells). Changes in the level of phosphorylated p38 MAPK (Δp38) in response to LPS stimulation were not statistically associated with changes in oestradiol levels (ΔE2). Multiple linear regression analyses adjusting for age revealed no associations between ΔE2 and Δp38 in PMNs (*p* = 0.813), monocytes (*p* = 0.363) or lymphocytes (*p* = 0.494).

### 3.7. Diapedesis Marker CD99 Expression and Association with Oestradiol Response

#### 3.7.1. Controls

There was no difference in CD99 expression on PMNs (*p* = 0.241), monocytes (*p* = 0.670) or lymphocytes (*p* = 0.326) between baseline and post-treatment timepoints. No associations were found between changes in CD99 expression (ΔCD99) on any of the cell subsets and the variation in oestradiol levels (ΔE2). Linear regression analyses adjusting for age revealed no predictive value of ΔCD99 for ΔE2 in PMNs (*p* = 0.909), monocytes (*p* = 0.960) or lymphocytes (*p* = 0.526). Age did not emerge as a covariate in any of the models (all *p* > 0.164).

#### 3.7.2. LPS Stimulation

No changes were found in CD99 expression for PMNs (*p* = 0.303), monocytes (*p* = 0.626) or lymphocytes (*p* = 0.626) between baseline and ovulation onset. Changes in CD99 expression (ΔCD99) in response to LPS stimulation did not correlate with changes in oestradiol levels (ΔE2), even when adjusted for age in multiple linear regression models. Age was not a covariate in any model.

### 3.8. Inflammatory Cytokines Expression and Association with Oestradiol Response

**Figure 3:**
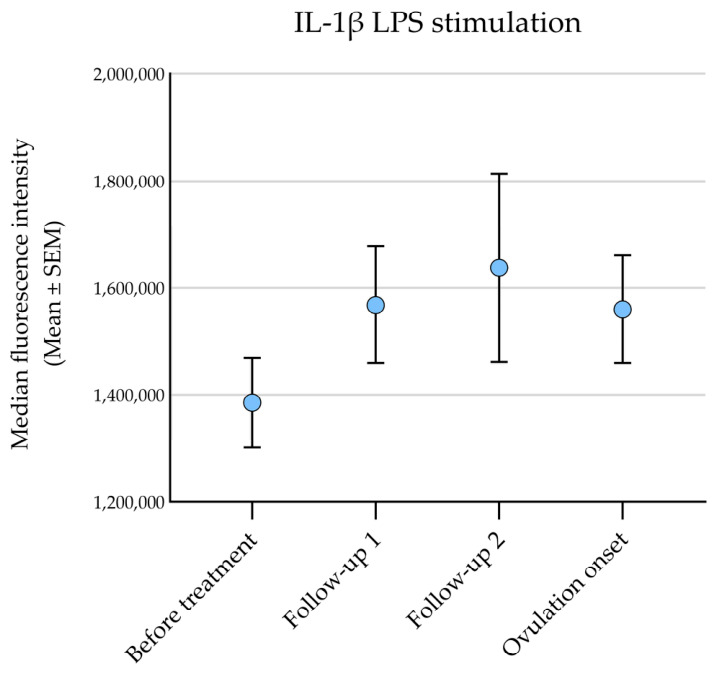
IL-1β expression throughout ovarian stimulation treatment after LPS stimulation. Comparable findings were obtained for all cytokines measured (IL-1β, IL-6, TNF-α and IL-10) across all TLR stimulation conditions tested, with no significant changes in cytokine production observed throughout ovarian hyperstimulation treatment (Wilcoxon matched-pairs signed-rank test). The only notable exception was the response to TLR2/6 stimulation (zymosan).

Analysis of cytokine release in whole blood revealed no differences in IL-1β, IL-6, IL-8, IL-10 or IL-12(p70) concentrations between the baseline (before treatment) and ovulation onset after stimulation for most of the tested TLR ligands (Figure 3, Figures S2, S4 and S6–S9). Specifically, for the controls, as well as for TLR1/2 (PAM3CSK4), TLR4 (LPS), TLR7/8 (R848) and TLR2/6 (Zymosan), our tests yielded non-significant *p*-values.

**Figure 4:**
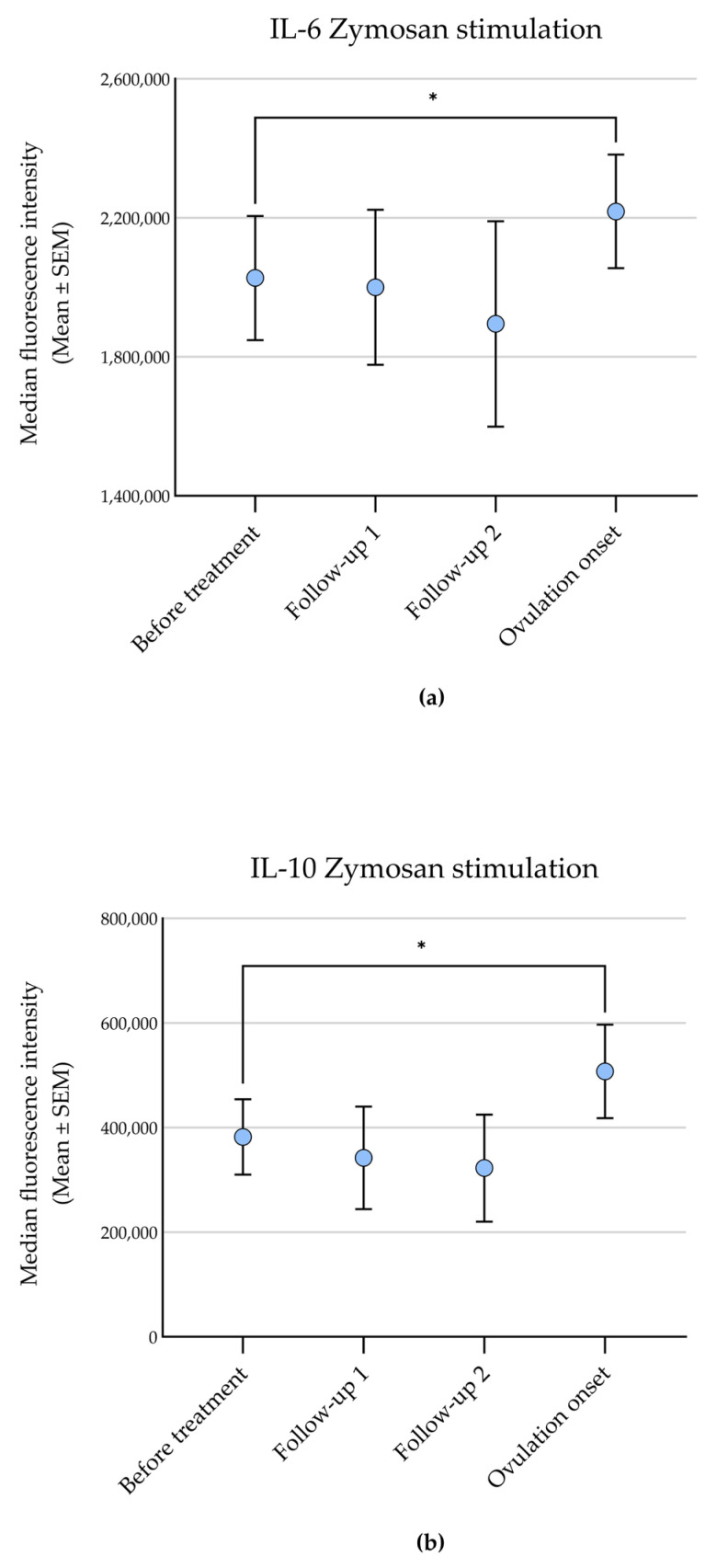
Changes in IL-6 and IL-10 cytokine production following TLR2/6 stimulation (zymosan) throughout ovarian stimulation treatment. Significant differences are indicated by an asterisk (*). Exact *p*-values are reported below. (**a**) IL-6 secretion after zymosan stimulation increased significantly between baseline (before treatment) and ovulation onset (*p* = 0.035, Wilcoxon matched-pairs signed-rank test, *n* = 16). Changes in IL-6 production were positively associated with variations in circulating oestradiol concentrations (ΔE2) after adjustment for age (*p* = 0.016, *n* = 16). (**b**) IL-10 secretion after zymosan stimulation also increased significantly between baseline and ovulation onset (*p* < 0.001, Wilcoxon matched-pairs signed-rank test, *n* = 16). However, no significant association was observed between changes in IL-10 production and ΔE2 levels after age adjustment (*p* = 0.101, *n* = 16).

An exception was observed for IL-6 and IL-10 in response to TLR2/6 stimulation (Zymosan), where a significant increase was detected between the baseline (before treatment) and ovulation onset (IL-6_ZYM: *p* = 0.035; IL-10_ZYM: *p* < 0.001) (Figure 4, Figures S3 and S6). Additionally, the regression analysis adjusting for age showed an association between the Δ of IL-6_ZYM and ΔE2 levels (*p* = 0.016). No such association was found for IL-10_ZYM (*p* = 0.101).

## 4. Discussion

In this study, we investigated X-linked innate immune responses during a progressive rise in circulating oestradiol levels. Consistent with our hypothesis, this hormonal surge did not alter most of these responses, as observed in patients with different numbers of sex chromosomes.

The impact of oestrogens on TLR2 and TLR4 expression has been reported but remains unresolved. Regarding protein expression, oestradiol has been shown to downregulate TLR2 in human monocytes, without affecting TLR4 [15], although these findings are limited to in vitro β-oestradiol exposure. At the transcriptional level, findings remain inconsistent; some studies report no effect of oestradiol [16,17], while others report downregulation [18] or even upregulation of *TLR2* mRNA, depending on the model [19,20], while *TLR4* mRNA expression appears unaffected [15,17,21]. Our study extends this literature by showing no modulation of TLR2 or TLR4 protein expression after in vivo exposure to high oestradiol levels.

**Figure 5:**
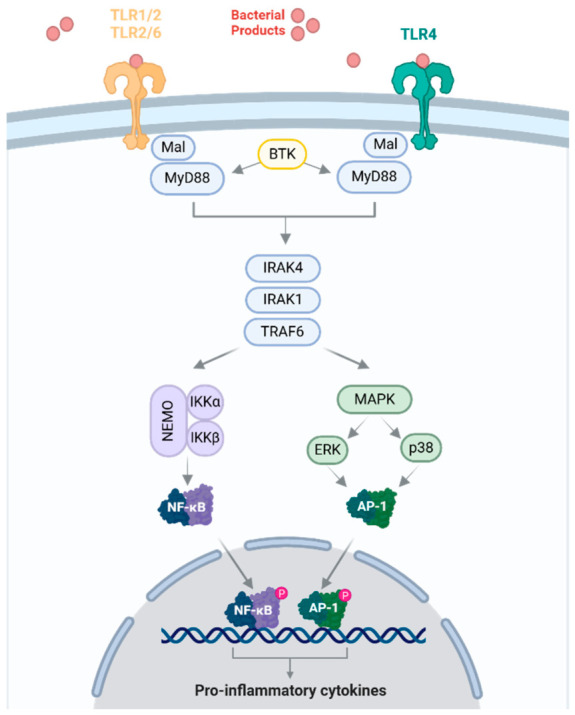
Activation of TLR2 or TLR4 initiates NF-κB phosphorylation, leading to NF-κB-regulated factor stimulation and triggering the p38 MAPK and ERK1/2 pathways. This process activates the AP-1 transcription factor, collectively driving the production of proinflammatory cytokines. Created in BioRender. Popotas, A. (2026) https://BioRender.com/450xgtf, based on papers from Takeda and Akira [25]; Asami and Shimizu [26]; Russell [27].

Activation of TLR2 or TLR4 during an inflammatory response initiates the phosphorylation of the NF-κB complex, thereby stimulating NF-κB-regulated factors [22,23] (Figure 5). This activation also triggers the p38 MAPK and ERK1/2 signalling pathways, which subsequently induce the activity of the AP-1 transcription factor [22,23]. The collective activation of NF-κB and AP-1 ultimately promotes the production of proinflammatory cytokines [24].

Prior research has suggested that high doses of oestrogen can downregulate NF-κB and Th1 responses in various cell types [25]. In vitro studies on human cord blood mononuclear cells and human monocytes further report that oestrogens inhibit the NF-κB pathway and do not affect the MAPK signalling pathway [15,17]. In our cohort, we examined the effects of in vivo high oestradiol levels on intracellular signalling pathways in peripheral blood leukocytes. Oestradiol showed only a weak inverse association with the level of phosphorylated NF-κB p65 in monocytes in controls, but not after LPS stimulation. The level of phosphorylated ERK1/2 declined during ovarian stimulation, but this was age-related rather than oestradiol-driven, and phosphorylated p38 MAPK remained unchanged. Together, these findings indicate that oestradiol exerts only a limited in vivo effect on TLR-dependent intracellular signalling.

**Figure 6:**
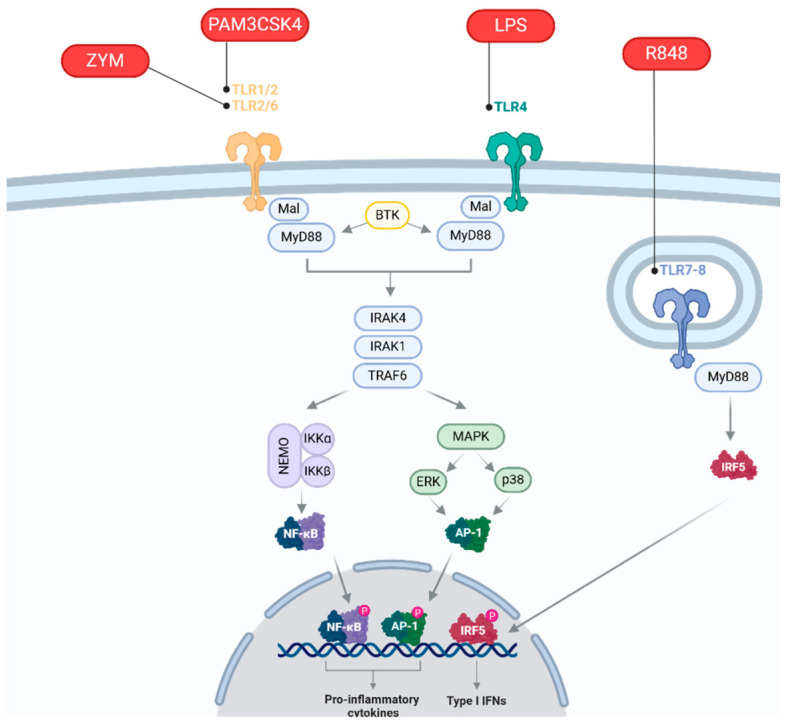
Schematic representation of TLR signalling pathways activated by different ligands: TLR4 with LPS, TLR1/2 with PAM3CSK4, TLR2/6 with Zymosan, and TLR7/8 with R848. Upon activation, the TIR domain of TLR2 and TLR4 interacts with MyD88, leading to the recruitment and activation of IRAK1, IRAK4 and TRAF6. This cascade induces the phosphorylation of the NF-κB complex and activates NF-κB-regulated factors, while also triggering p38 MAPK and ERK1/2 pathways, which enhance AP-1 activity. Together, NF-κB and AP-1 drive the production of proinflammatory cytokines. TLR7 and TLR8 are involved in IFN expression through TRIF-independent pathways, potentially mediated by IRF-5 or the MyD88–IRAK1–TRAF6 axis, although the precise mechanisms remain to be clarified. Created in BioRender. Popotas, A. (2026) https://BioRender.com/ck7jmwo.

We also analysed the influence of oestrogens on TLR activation by measuring cytokine production in response to TLR agonists. In whole blood during ovarian hyperstimulation treatment, oestradiol had minimal impact on cytokine expression. Across a range of TLR ligands—including TLR1/2, TLR4 and TLR7/8 (Figure 6)—cytokine secretion remained unchanged between baseline and ovulation onset (Figure 3, Figures S2, S4 and S6–S9). A selective effect was observed only after TLR2/6 stimulation (Zymosan): IL-6 secretion correlated with oestradiol variation, while IL-10 secretion increased significantly but lost significance after age adjustment (Figure 4, Figures S3 and S6).

Overall, our results differ from some in vitro reports of oestradiol-mediated decrease or enhancement of inflammatory cytokines [17,18,26,27] and align with clinical findings showing minimal cytokine modulation during hormone exposure [28]. Notably, studies in postmenopausal women receiving menopausal hormone therapy have similarly reported limited effects of oestradiol replacement on inflammatory cytokine responses, further supporting the notion that circulating oestradiol levels may exert only modest effects on innate immune function in vivo (Table 1).

Earlier research highlights that sex differences in TLR responsiveness may be more strongly linked to X-chromosome dosage than to sex steroids. Upon TLR7 stimulation, females exhibit higher baseline IFN levels compared to males [29], even before puberty [30]. This sex difference has been associated with the number of X chromosomes, as individuals with two X chromosomes demonstrate higher IFN production following TLR7 stimulation, regardless of sex steroid levels [30]. The differential expression and function of TLR7, with a female predominance, was attributed to the X chromosome, as the *TLR7* gene escapes X inactivation in plasmacytoid dendritic cells, monocytes and B cells [12,31]. Evidence from individuals with Klinefelter syndrome further supports this X chromosome-driven mechanism [5,12]. The production of inflammatory cytokines in response to TLR ligands was not influenced by 17-β oestradiol levels, suggesting a limited role for oestrogens in these immune responses [32]. In agreement with these previous reports, the marked increase in circulating 17β-oestradiol achieved during controlled ovarian hyperstimulation did not significantly alter TLR7/8-mediated immune responses in our cohort. These findings are consistent with the notion that the well-described sex differences in TLR7 responsiveness are unlikely to be explained solely by variations in circulating oestrogen concentrations. However, as no genomic or transcriptomic analyses were performed in the present study, the relative contribution of X-linked mechanisms cannot be directly assessed in our study [14] and warrants further investigation.

The CD99 gene, located near the boundary of the PAR1 and X-linked segments of the X chromosome, encodes a surface marker essential for monocyte migration across endothelial barriers. Although PAR1 genes are generally expressed equally in both sexes, spreading of X-chromosome inactivation into this boundary may partially silence CD99 in females, leading to a lower proportion of CD99-expressing monocytes [8]. The underlying mechanism remains unclear. In our study, oestrogen was excluded as a significant regulator of CD99 expression in leukocytes, as no changes were observed in relation to oestradiol variation.

A significant strength of this study is its in vivo design, investigating immune responses during controlled ovarian hyperstimulation, a physiological context in which oestradiol levels rise from physiological to supraphysiological concentrations. This approach enabled us to assess the effects of oestrogen on TLR signalling and cytokine production directly in whole blood, thereby preserving the complexity of cellular interactions and avoiding artefacts from isolated cell cultures. Another strength is the multimodal analysis of receptors, intracellular signalling and cytokine secretion, which together provide a comprehensive evaluation of TLR function.

Limitations include the relatively small sample size, which may have hindered detection of subtle effects, and inter-individual variability inherent to in vivo studies, in contrast to experimental models where environmental and temporal parameters can be tightly controlled.

## 5. Conclusions

Our findings suggest that oestradiol probably exerts only a limited influence on innate immune pathways, particularly mediated by TLR pathways, challenging the assumption that sex steroids are the primary drivers of sex-specific immunity. While the present study was not designed to directly investigate genetic mechanisms, our results support the growing body of evidence indicating that factors other than sex steroid concentrations, including X-linked immune pathways, may contribute substantially to immune sexual dimorphism. Further ex vivo and in vivo studies are needed to clarify the contribution of sex steroids, ideally complemented by comprehensive gene expression analyses of TLR2- and TLR4-related pathways. Extending this research to individuals undergoing hormone therapy or with steroid resistance syndromes may yield additional valuable perspectives on the mechanisms shaping sex-specific immune responses.

## Figures and Tables

**Table 1:** Reported effects of oestradiol on cytokine production across in vitro and in vivo models (↓ = decrease; ↑ = increase).

Reference	Model/Population	Experimental Context	Reported Effect on Cytokines
Giannoni E, Guignard L, Knaup Reymond M, Perreau M, Roth-Kleiner M, Calandra T et al. [17]	Human cord blood mononuclear cells (in vitro)	Stimulation + oestradiol	↓ TNF, ↓ IL-6 (protein & mRNA)
Souza CLS e, Barbosa CD, Coelho HILN, Santos Júnior MN, Barbosa EN, Queiroz ÉC et al. [18]	Human peripheral blood monocytes (HPBM, in vitro)	Bacterial stimulation + exogenous or endogenous oestradiol	↓ IL-1β, ↓ IL-6, ↓ TNF-α, ↓ GM-CSF; ↑ IL-10, ↑ IL-12, ↑ IL-23, ↑ IL-27
Asai K, Hiki N, Mimura Y, Ogawa T, Unou K, Kaminishi M. [26]	Human PBMCs (in vitro)	LPS + oestradiol	↓ TNF-α
S. Lashkari B, Anumba DOC. [27]	Human ectocervical epithelial cells (in vitro)	LPS + oestradiol	↑ IL-1β, ↑ IL-6, ↑ IL-8, ↑ IFN-γ
Rogers A, Eastell R. [28]	Menopausal women (in vivo, HRT)	Hormone replacement therapy	No effect on IL-6, IL-1ra, IL-1α, TNF-α

## Data Availability

The original contributions presented in this study are included in the article/Supplementary Materials. Further inquiries can be directed to the corresponding author.

## Supplementary Materials

The following supporting information can be downloaded at: https://www.mdpi.com/article/10.3390/cells15141257/s1. Figure S1: Validation of whole blood responsiveness following ex vivo stimulation; Figures S2–S9: Longitudinal assessment of cytokine responses during controlled ovarian hyperstimulation; Table S1: Descriptive statistics—Categorical variables; Table S2: Descriptive statistics—Continuous variables.

## Abbreviations

The following abbreviations are used in this manuscript:

A488	Alexa Fluor 488
AP-1	Activator Protein 1
APC	Allophycocyanin
BTK	Bruton’s Tyrosine Kinase
CD	Cluster of Differentiation
Cy7	Cyanine 7
E2	Oestradiol
ERK1/2	Extracellular Signal-Regulated Kinases ½
FACS	Fluorescence-Activated Cell Sorting
FBS	Fetal Bovine Serum
FITC	Fluorescein Isothiocyanate
FSH	Follicle-Stimulating Hormone
GM-CSF	Granulocyte-Macrophage Colony-Stimulating Factor
GnRH	Gonadotropin-Releasing Hormone
hCG	Human Chorionic Gonadotropin
HIV	Human Immunodeficiency Virus
HPBM	Human Peripheral Blood Monocytes
HRT	Hormone Replacement Therapy
IFN	Interferon
IFN-α2	Interferon alpha 2
IFN-γ	Interferon gamma
IL	Interleukin
IRAK	Interleukin-1 Receptor-Associated Kinase
IRF-5	Interferon Regulatory Factor 5
IVF	In Vitro Fertilisation
LPS	Lipopolysaccharide
mAbs	Monoclonal Antibodies
MAPK	Mitogen-Activated Protein Kinase
MyD88	Myeloid Differentiation Primary Response 88
NF-κB	Nuclear Factor-kappa B
PAR1	Pseudoautosomal Region 1
PBS	Phosphate-Buffered Saline
PBMCs	Peripheral Blood Mononuclear Cells
PCOS	polycystic ovary syndrome
PE	Phycoerythrin
PMNs	Polymorphonuclear Cells
R848	Resiquimod
RPMI	Roswell Park Memorial Institute
SSC	Side Scatter
TLR	Toll-Like Receptor
TNF-α	Tumour Necrosis Factor alpha
TRAF6	TNF Receptor-Associated Factor 6
TRIF	TIR-domain-containing adapter-inducing interferon-β
ZYM	Zymosan
