# Supplementary material for: Is Oestradiol a Key Player in the Sex Differences in Innate Immunity Through Toll-like Receptor Activation?
Source: Cells. 2026 Jul 13;15(14):1257. doi: 10.3390/cells15141257 (PMC13406721; doi:10.3390/cells15141257)
Supplement: Supplementary file 1 [file cells-15-01257-s001.zip › cells-4386811-supplementary.pdf]

Supplementary Data

Cytokine secretion following whole blood stimulation

Before ovarian hyperstimulation treatment | On ovulation triggering

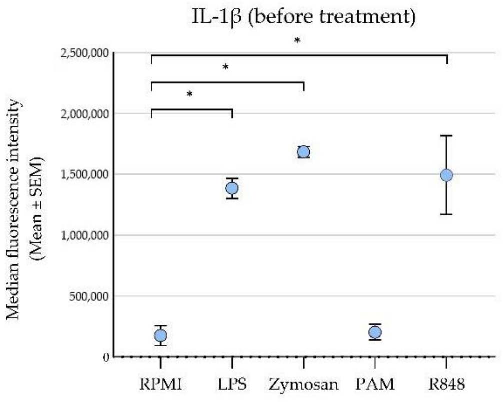

(a)

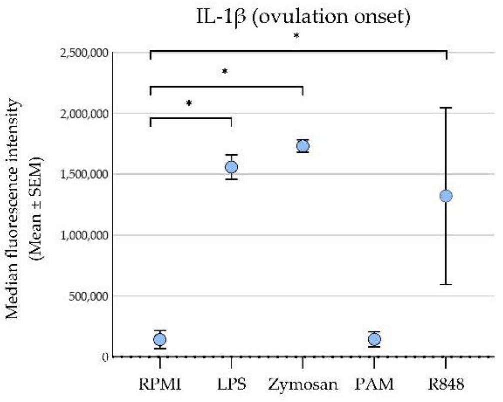

(b)

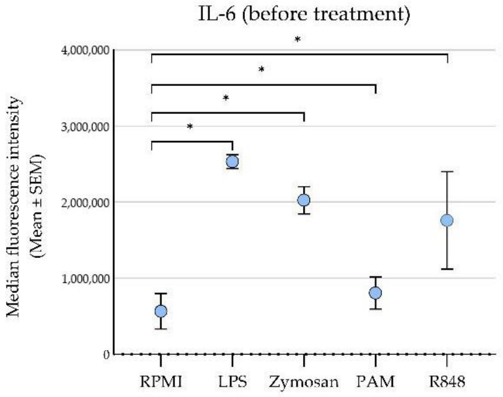

(c)

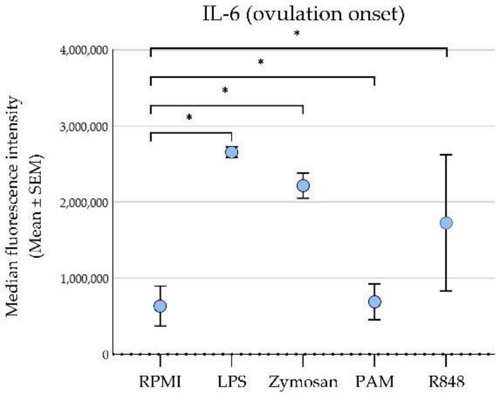

(d)

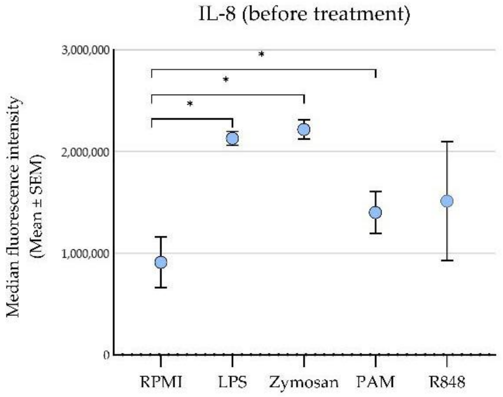

(e)

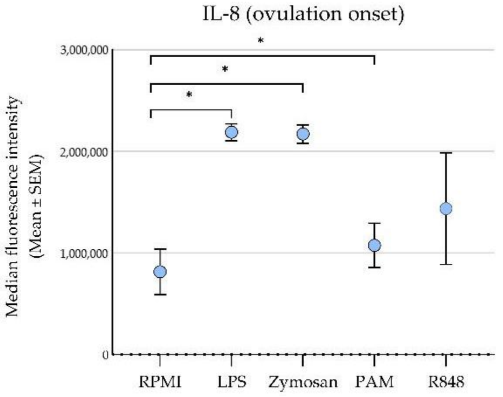

(f)

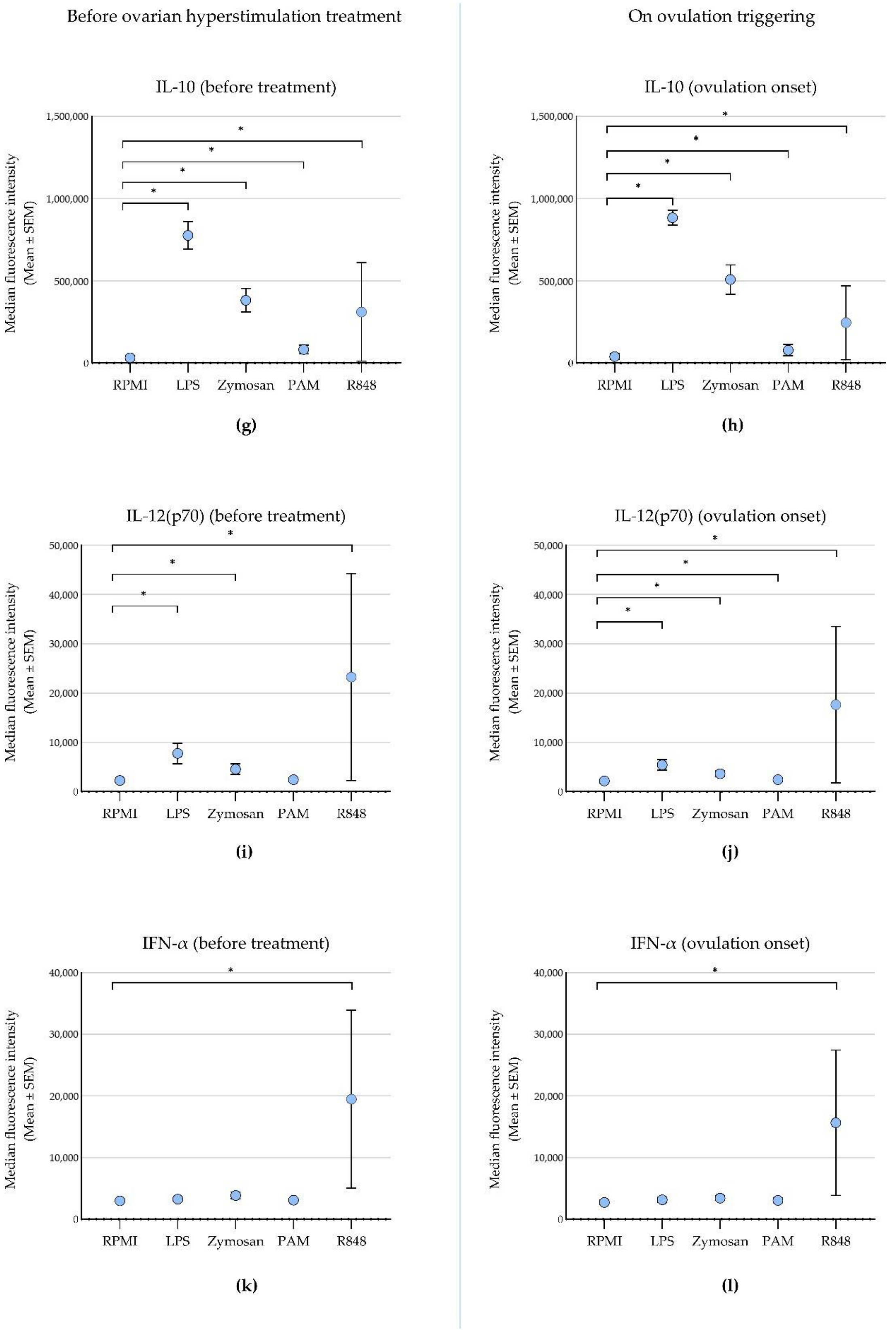

Before ovarian hyperstimulation treatment
On ovulation triggering
IL-10 (before treatment)
IL-10 (ovulation onset)
IL-12(p70) (before treatment)
IL-12(p70) (ovulation onset)
IFN-α (before treatment)
IFN-α (ovulation onset)
Median fluorescence intensity (Mean ± SEM)
0
500,000
1,000,000
1,500,000
10,000
20,000
30,000
40,000
50,000
*
RPMI
LPS
Zymosan
PAM
R848
(g)
(h)
(i)
(j)
(k)
(l)

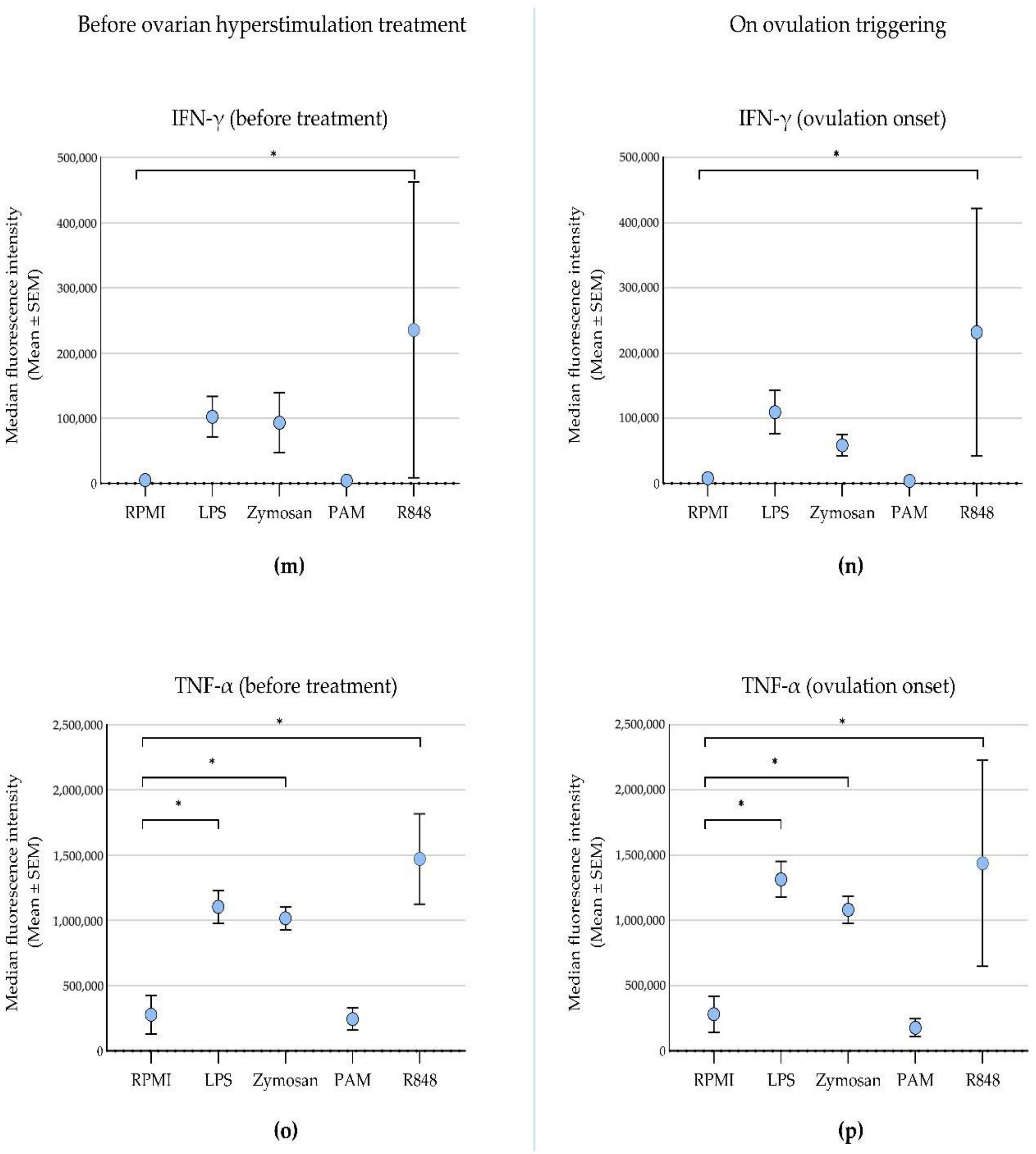

Figure S1. Validation of whole blood responsiveness following ex vivo stimulation. Whole blood samples collected before controlled ovarian hyperstimulation treatment (a, c, e, g, i, k, m, o) and at ovulation triggering (b, d, f, h, j, l, n, p) were stimulated with RPMI medium alone (negative control), lipopolysaccharide (LPS), zymosan, PAM3CSK4 (PAM), or R848. Secretion of IL-1β (a,b), IL-6 (c,d), IL-8 (e,f), IL-10 (g,h), IL-12(p70) (i,j), IFN-α (k,l), IFN-γ (m,n), and TNF-α (o,p) was measured and expressed as median fluorescence intensity (MFI; mean ± SEM). These analyses were performed to assess the responsiveness of circulating immune cells to different pattern-recognition receptor agonists and to validate the effectiveness of the stimulation conditions. Statistical comparisons were performed between each stimulation condition and the RPMI control using paired Wilcoxon signed-rank tests

(n = 16). Significant differences are indicated by asterisks (*$p < 0.05$). Exact p-values are reported below.

(a) IL-1β before treatment: LPS ($p < 0.0001$), zymosan ($p < 0.0001$), and R848 ($p = 0.0001$) significantly increased IL-1β secretion compared with RPMI, whereas PAM was not significant ($p = 0.0833$).

(b) IL-1β at ovulation triggering: LPS ($p < 0.0001$), zymosan ($p < 0.0001$), and R848 ($p = 0.0004$) significantly increased IL-1β secretion compared with RPMI, whereas PAM was not significant ($p = 0.0833$).

(c) IL-6 before treatment: LPS ($p = 0.0003$), zymosan ($p = 0.0012$), PAM ($p = 0.0151$), and R848 ($p = 0.0084$) significantly increased IL-6 secretion compared with RPMI.

(d) IL-6 at ovulation triggering: LPS ($p = 0.0002$), zymosan ($p = 0.0012$), PAM ($p = 0.0215$), and R848 ($p = 0.0413$) significantly increased IL-6 secretion compared with RPMI.

(e) IL-8 before treatment: LPS ($p = 0.0009$), zymosan ($p = 0.0009$), and PAM ($p = 0.0067$) significantly increased IL-8 secretion compared with RPMI, whereas R848 was not significant ($p = 0.0637$).

(f) IL-8 at ovulation triggering: LPS ($p = 0.0003$), zymosan ($p = 0.0015$), and PAM ($p = 0.0084$) significantly increased IL-8 secretion compared with RPMI, whereas R848 was not significant ($p = 0.0946$).

(g) IL-10 before treatment: LPS ($p < 0.0001$), zymosan ($p = 0.0002$), PAM ($p = 0.0034$), and R848 ($p = 0.0061$) significantly increased IL-10 secretion compared with RPMI.

(h) IL-10 at ovulation triggering: LPS ($p < 0.0001$), zymosan ($p < 0.0001$), PAM ($p = 0.0043$), and R848 ($p = 0.0215$) significantly increased IL-10 secretion compared with RPMI.

(i) IL-12(p70) before treatment: LPS ($p = 0.0001$), zymosan ($p < 0.0001$), and R848 ($p = 0.0010$) significantly increased IL-12(p70) secretion compared with RPMI, whereas PAM was not significant ($p = 0.2078$).

(j) IL-12(p70) at ovulation triggering: LPS ($p < 0.0001$), zymosan ($p < 0.0001$), PAM ($p = 0.0067$), and R848 ($p = 0.0002$) significantly increased IL-12(p70) secretion compared with RPMI.

(k) IFN-α before treatment: R848 significantly increased IFN-α secretion ($p < 0.0001$), whereas PAM was not significant ($p = 0.5614$).

(l) IFN-α at ovulation triggering: R848 ($p < 0.0001$) and PAM ($p = 0.0125$) significantly increased IFN-α secretion compared with RPMI.

(m) IFN-γ before treatment: R848 significantly increased IFN-γ secretion (p = 0.0002), whereas PAM was not significant (p = 0.5245).

(n) IFN-γ at ovulation triggering: R848 significantly increased IFN-γ secretion (p = 0.0005), whereas PAM was not significant (p = 0.2524).

(o) TNF-α before treatment: LPS (p = 0.0012), zymosan (p = 0.0009), and R848 (p = 0.0003) significantly increased TNF-α secretion compared with RPMI, whereas PAM was not significant (p = 0.3028).

(p) TNF-α at ovulation triggering: LPS (p = 0.0001), zymosan (p = 0.0006), and R848 (p = 0.0009) significantly increased TNF-α secretion compared with RPMI, whereas PAM was not significant (p = 0.0833).

Table S1. Descriptive statistics – Categorical variables

Categorical variables

**Pre_stimulation_treatment**					
		Frequency	Percentage	Valid percentage	Cumulative percentage
Valid	No pre-stimulation treatment	11	68,8	68,8	68,8
	Progynova	5	31,3	31,3	100,0
	Total	16	100,0	100,0	

**Lh_Rh_Modulator**					
		Frequency	Percentage	Valid percentage	Cumulative percentage
Valid	1	16	100,0	100,0	100,0

**Stimulation_treatement**					
		Frequency	Percentage	Valid percentage	Cumulative percentage
Valide	Menopur	5	31,3	31,3	31,3
	Ovaleap	10	62,5	62,5	93,8
	Gonal-F	1	6,3	6,3	100,0
	Total	16	100,0	100,0	

**Ovulation_onset**					
		Frequency	Percentage	Valid percentage	Cumulative percentage
Valid	Recombinant hCG	11	68,8	68,8	68,8
	Triptorelin	1	6,3	6,3	75,0
	Recombinant hCG + Triptorelin	3	18,8	18,8	93,8
	No stimulaion	1	6,3	6,3	100,0
	Total	16	100,0	100,0	

Table S2. Descriptive statistics – Continuous variables

Age					
N	Minimum	Maximum	Mean	Standard error	Standard deviaton
16	26	39	33,19	1,085	4,339

IL-1β

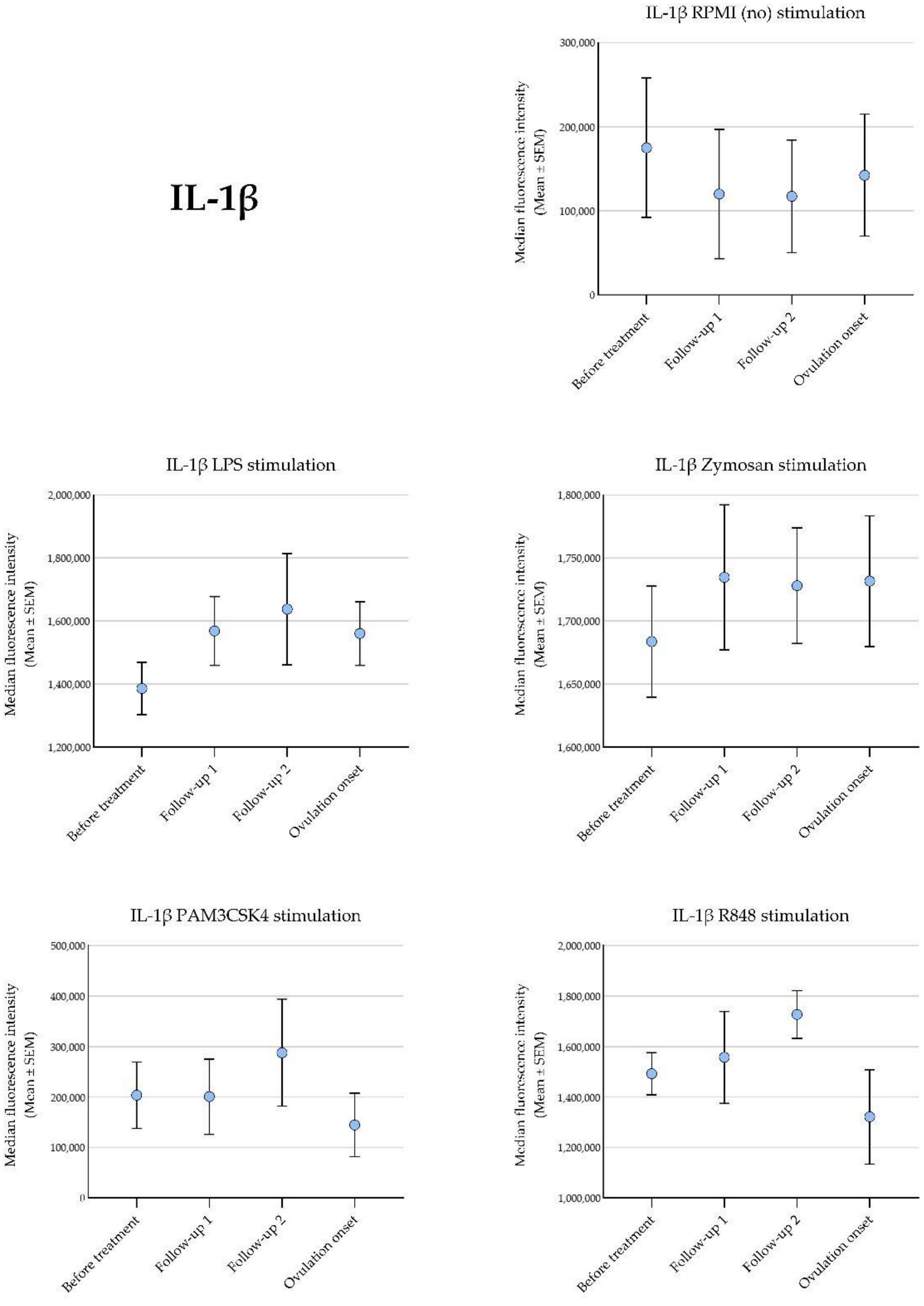

Figure S2. Longitudinal assessment of IL-1β responses during controlled ovarian hyperstimulation. IL-1β production following stimulation with RPMI, LPS, ZYM, PAM, or R848 was evaluated before treatment (baseline), at follow-up 1, follow-up 2, and on the day of ovulation induction. Results are presented as mean ± SEM. Statistical comparisons between time points were performed using the Wilcoxon signed-rank test.

IL-6

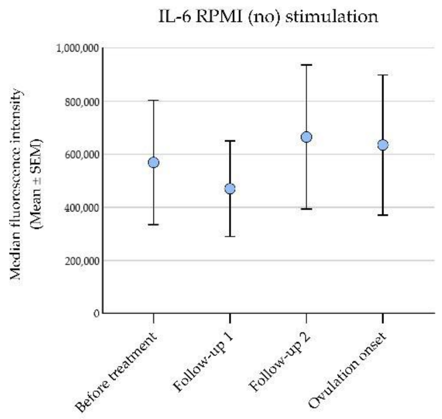

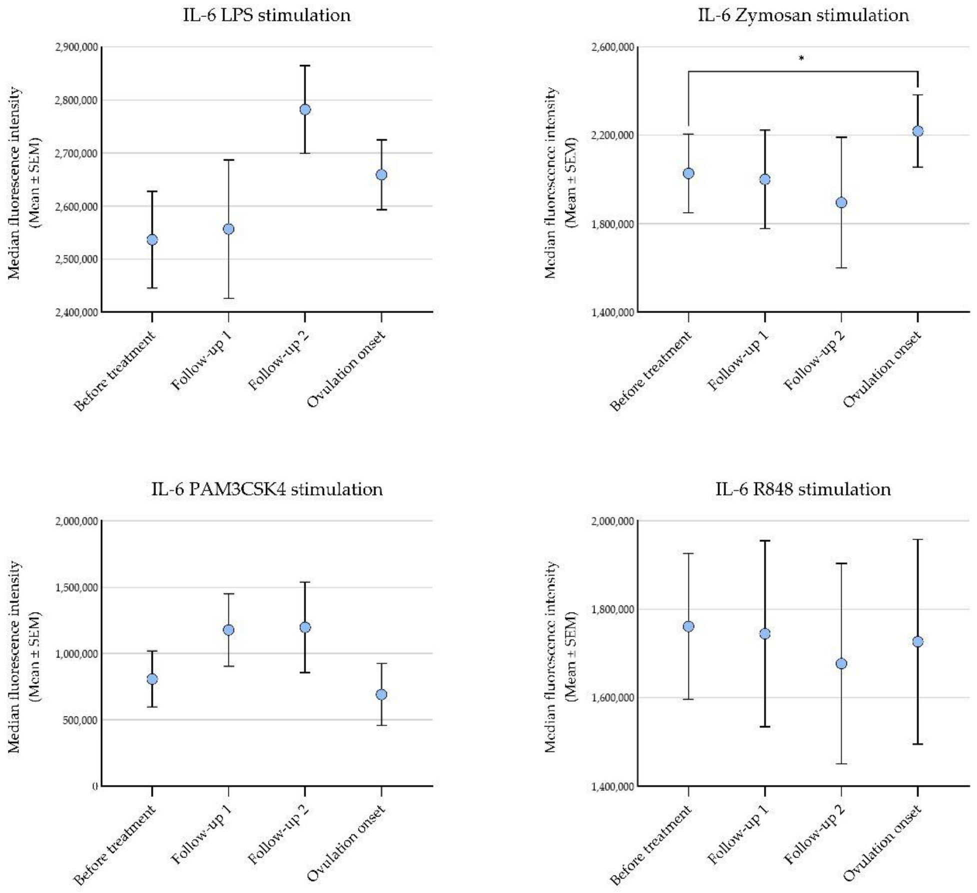

Figure S3. Longitudinal assessment of IL-6 responses during controlled ovarian hyperstimulation. IL-6 production following stimulation with RPMI, LPS, ZYM, PAM, or R848 was evaluated before treatment (baseline), at follow-up 1, follow-up 2, and on the day of ovulation induction. Results are presented as mean ± SEM. Statistical comparisons between time points were performed using the Wilcoxon signed-rank test. Asterisks indicate statistically significant differences only ($p < 0.05$).

IL-8

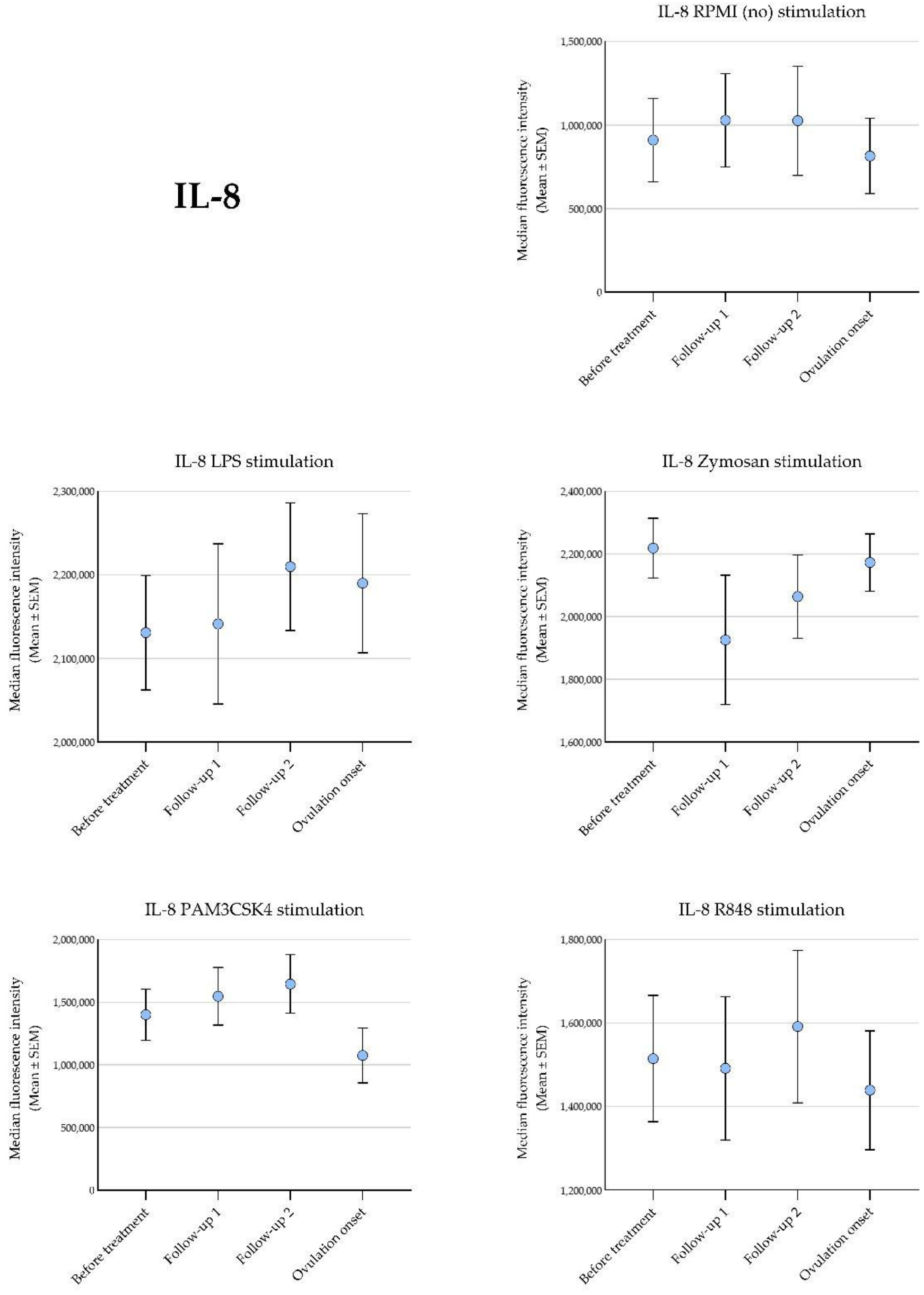

Figure S4. Longitudinal assessment of IL-8 responses during controlled ovarian hyperstimulation. IL-8 production following stimulation with RPMI, LPS, ZYM, PAM, or R848 was evaluated before treatment (baseline), at follow-up 1, follow-up 2, and on the day of ovulation induction. Results are presented as mean ± SEM. Statistical comparisons between time points were performed using the Wilcoxon signed-rank test.

IL-10

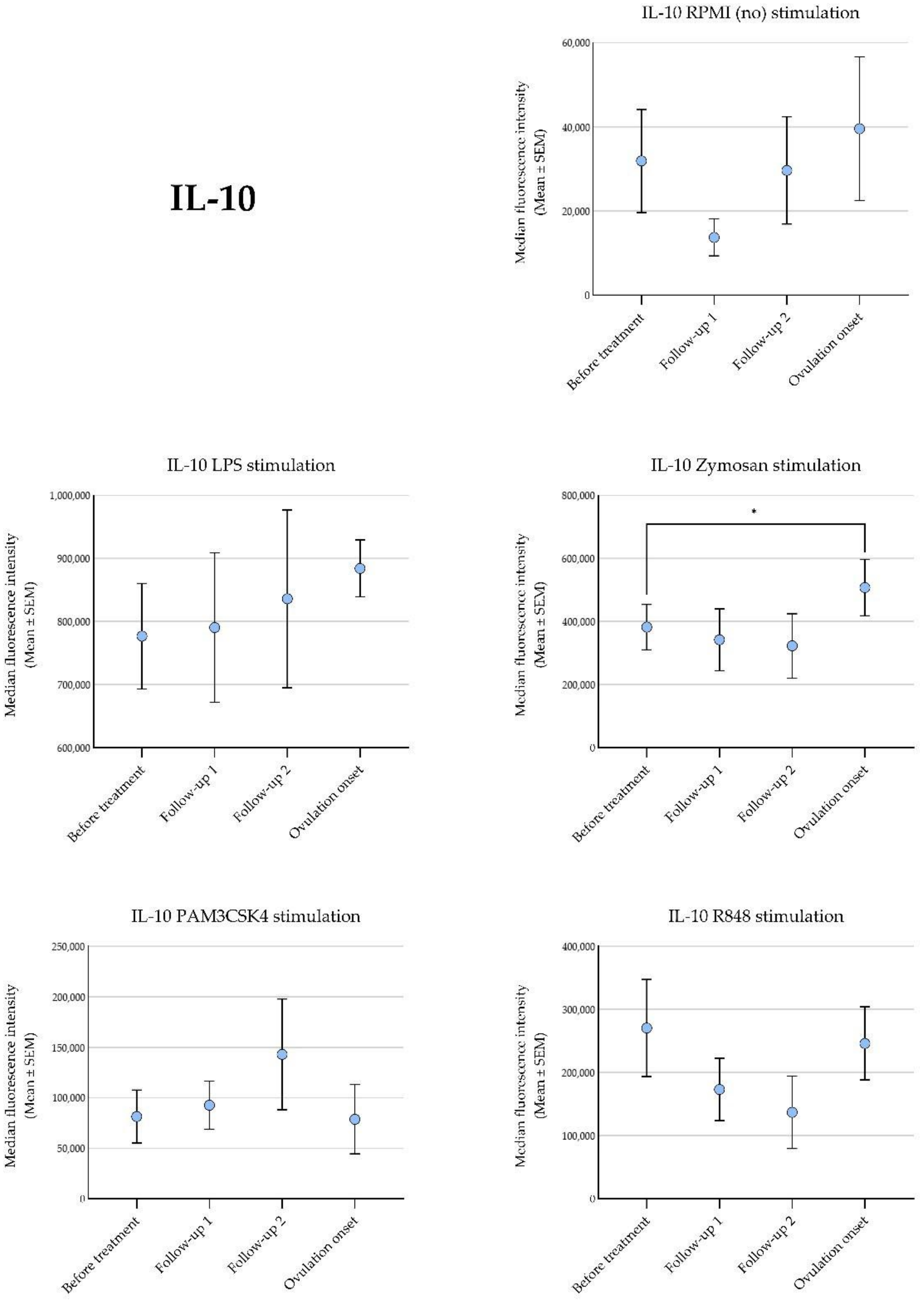

Figure S5. Longitudinal assessment of IL-10 responses during controlled ovarian hyperstimulation. IL-10 production following stimulation with RPMI, LPS, ZYM, PAM, or R848 was evaluated before treatment (baseline), at follow-up 1, follow-up 2, and on the day of ovulation induction. Results are presented as mean ± SEM. Statistical comparisons between time points were performed using the Wilcoxon signed-rank test. Asterisks indicate statistically significant differences only ($p < 0.05$).

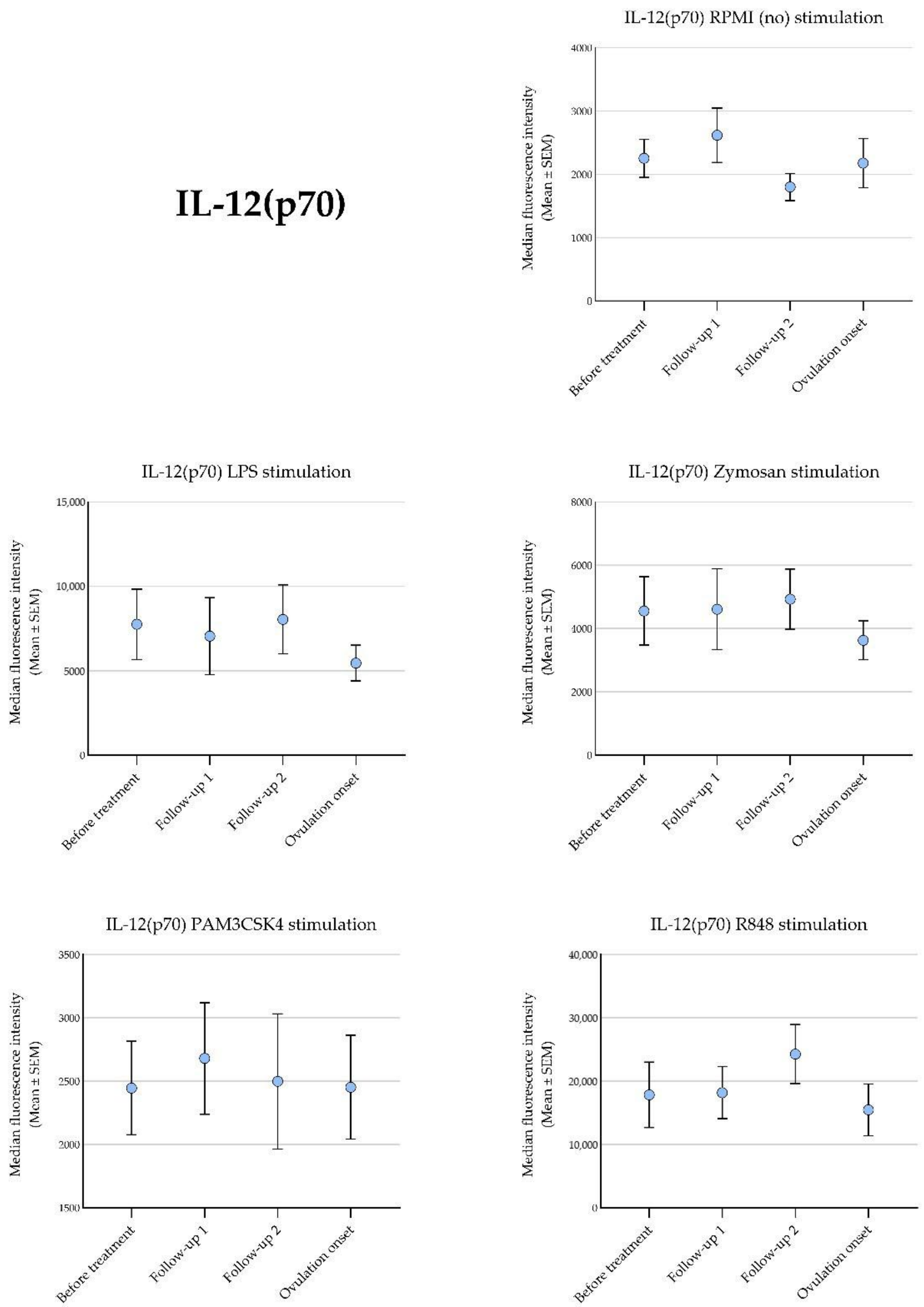

Figure S6. Longitudinal assessment of IL-12(p70) responses during controlled ovarian hyperstimulation. IL-12(p70) production following stimulation with RPMI, LPS, ZYM, PAM, or R848 was evaluated before treatment (baseline), at follow-up 1, follow-up 2, and on the day of ovulation induction. Results are presented as mean ± SEM. Statistical comparisons between time points were performed using the Wilcoxon signed-rank test.

IFN-α2

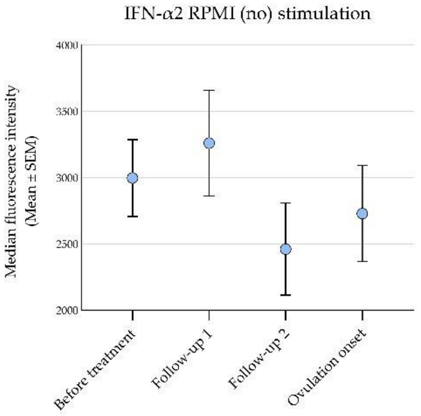

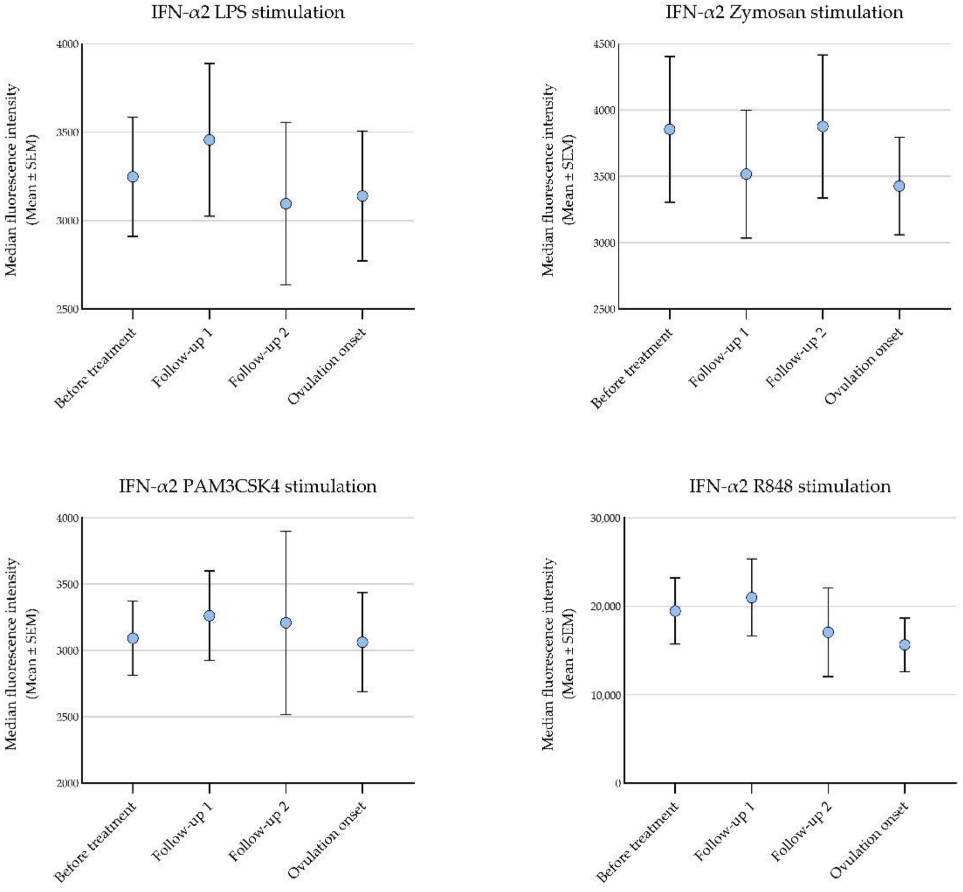

Figure S7. Longitudinal assessment of IFN-α responses during controlled ovarian hyperstimulation. IFN-α production following stimulation with RPMI, LPS, ZYM, PAM, or R848 was evaluated before treatment (baseline), at follow-up 1, follow-up 2, and on the day of ovulation induction. Results are presented as mean ± SEM. Statistical comparisons between time points were performed using the Wilcoxon signed-rank test.

IFN-γ

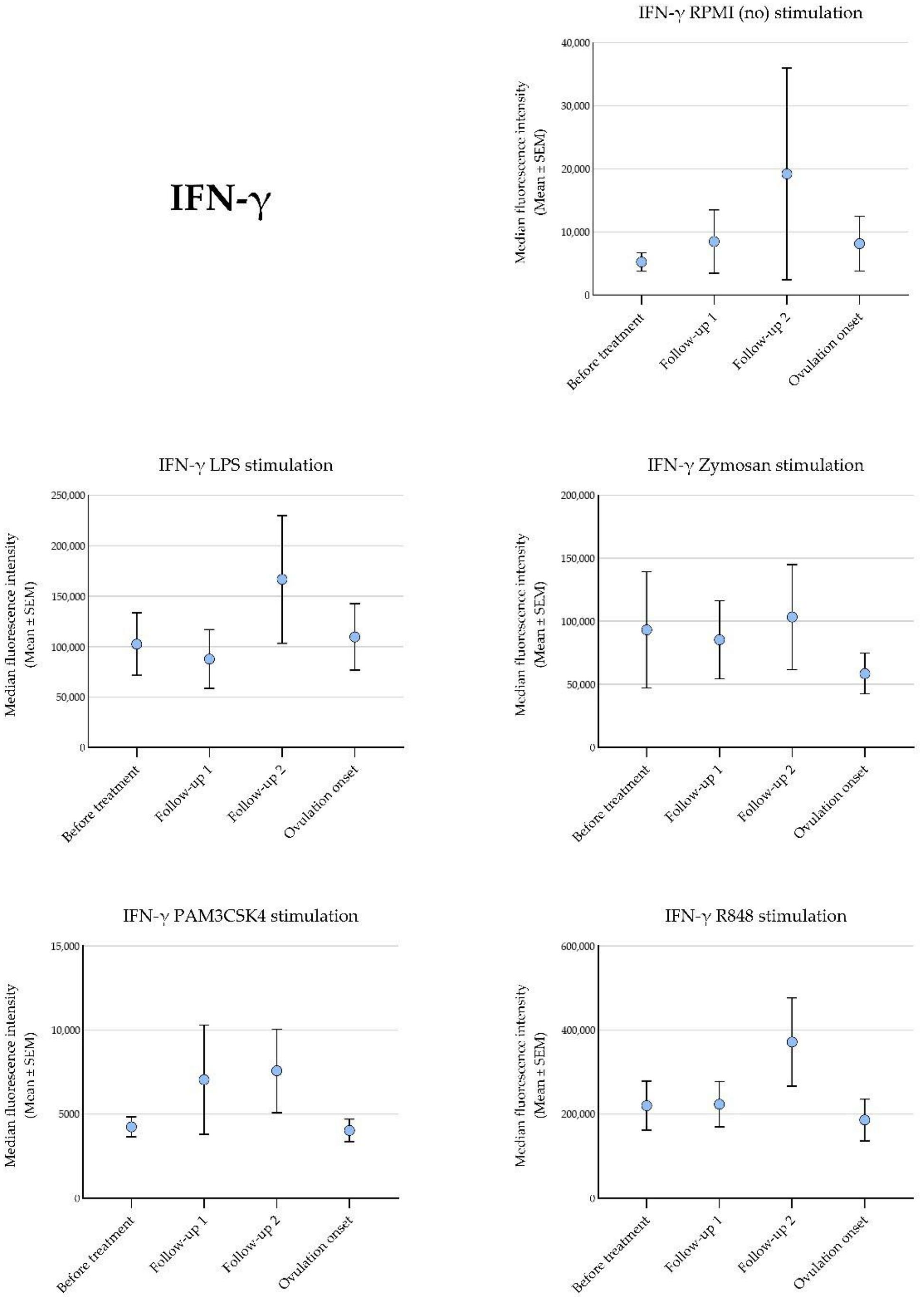

Figure S8. Longitudinal assessment of IFN-γ responses during controlled ovarian hyperstimulation. IFN-γ production following stimulation with RPMI, LPS, ZYM, PAM, or R848 was evaluated before treatment (baseline), at follow-up 1, follow-up 2, and on the day of ovulation induction. Results are presented as mean ± SEM. Statistical comparisons between time points were performed using the Wilcoxon signed-rank test.

TNF-α

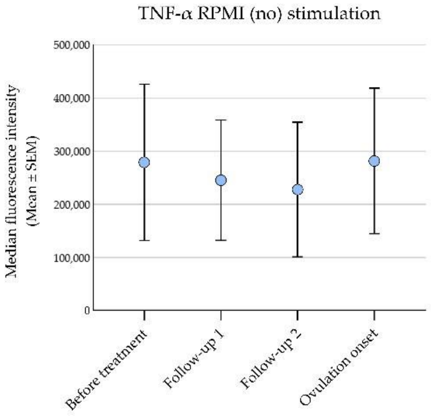

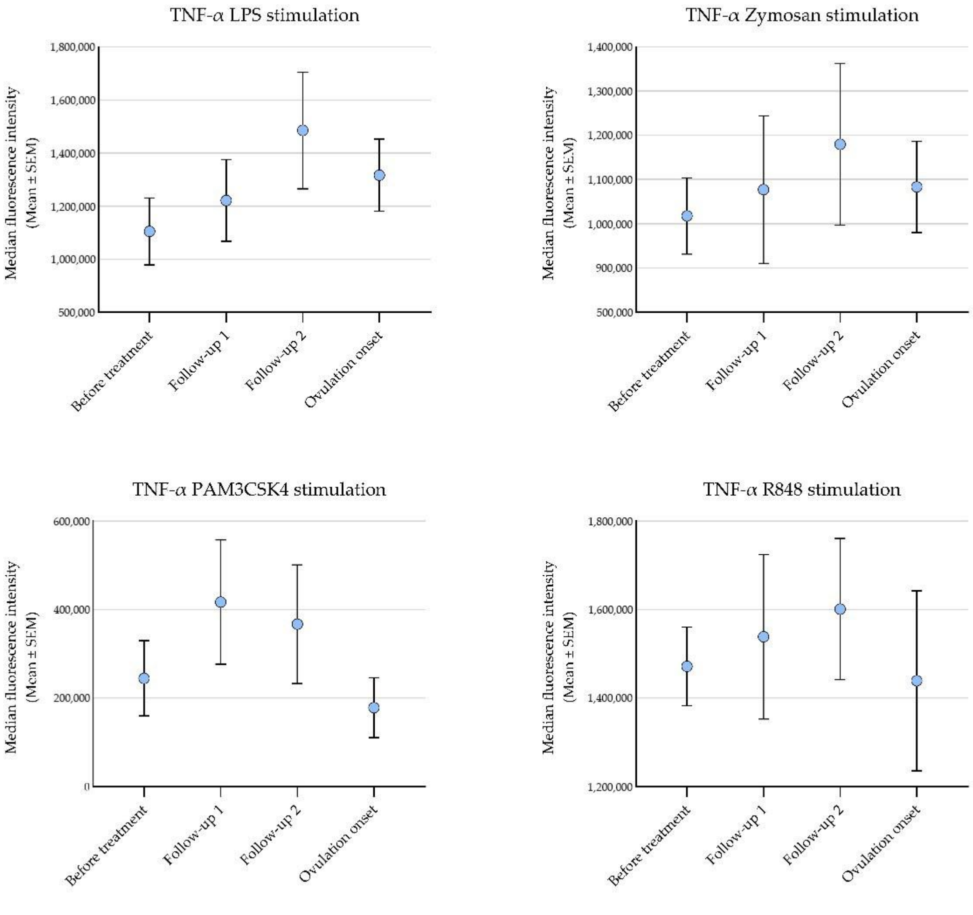

Figure S9. Longitudinal assessment of TNF-α responses during controlled ovarian hyperstimulation. TNF-α production following stimulation with RPMI, LPS, ZYM, PAM, or R848 was evaluated before treatment (baseline), at follow-up 1, follow-up 2, and on the day of ovulation induction. Results are presented as mean ± SEM. Statistical comparisons between time points were performed using the Wilcoxon signed-rank test.
